# Report on the Prevalence of Diseases, in the City of Chicago, from April 1st, to June 30th, 1866

**Published:** 1866-08

**Authors:** N. S. Davis

**Affiliations:** Committee


ARTICLE XXVIII.

### REPORT ON THE PREVALENCE OF DISEASES, IN THE CITY OF CHICAGO, FROM APRIL 1ST, TO JUNE 30TH, 1866.

BY N. S. DAVIS, M.D., Committee.

Read before the Chicago Medical Society, July 6th, 1866.

During the three months ending June 30th, 1866, the general sanitary condition of this city has been better than during the same part of the two preceding years. The months of April and May were cool and comparatively dry. The adoption of a scavenger system, though very imperfect, and the extra efforts to clean the street gutters and alleys earlier than usual, has made a perceptible improvement in the physical condition of the city. Still there are many things injurious to the public health that remain untouched, but they have been so often alluded to in former reports, that further enumeration is unnecessary at present.

During the past three months, the hydrant water for the city has been in comparatively good condition, and until the middle of June, the river was not notably offensive. Since the last-named period, the North Branch has been emitting a very offensive odor, and the South Branch, above 12th Street, has not been much better.

The atmospheric conditions during the months of April and May were highly favorable to health. The electricity was abundant, the moisture less than usual for the season, and the prevalent winds from the north, north-east, and west, rendering the atmosphere cool and invigorating to animal functions.

The only exception to this general condition, was the 19th of May, when a direct south wind brought a current of hot, damp atmosphere, extremely oppressive, but which ended in slight showers in the evening, with a change in the wind to the north, and decided coolness on the morning of the 20th. The same general conditions continued until the morning of the 8th of June, which was ushered in with a wind from the south, and continued hot, sultry, and very oppressive all day. Showers of rain fell in the evening. On the morning of the 9th, the wind blew from the west; and on the 10th, from the north and north-east, and both days were cool and bracing. During the morning of the 11th, the wind again changed to the south, and the afternoon became very hot and oppressive, with slight showers in the evening.

During the last three days, I have met with seven severe cases of dysentery, and several slighter ones, in different parts of the city. These constitute the first notable indications of the prevalence of bowel affections this summer.

The night of the 11th and all the 12th, the atmosphere continued hot, damp, and oppressive, with copious showers in the afternoon. Attacks of diarrhœa and dysentery increased rapidly, especially in children. One very severe case of cholera-morbus, in an adult, occurred during the night of the 11th, at 212 Kankakee Avenue, and another on West Harrison, near Morgan Street. Both were accompanied by cramps in the extremities and rice-water discharges, but neither proved fatal.

During the 13th, the wind was from the west, and the atmosphere cooler and dryer. The 14th, wind, in the morning, south, warm, and damp, but changed to S.W., with showers in the afternoon. During the 15th, 16th, 17th, 18th, and 19th, the wind was north and north-west, with the atmosphere dry, clear, and cool enough to make an overcoat comfortable. During these days, I saw only two new cases of cholera-morbus, but cases of dysentery continued to multiply, both in children and adults. On close inquiry, however, nearly all the cases began between the 11th and 14th.

On the 20th, wind again changed to south, and the afternoon very hot, oppressive, but clear. 21st, same, with showers and some lightning in the afternoon. From the 22d to the 26th, the prevalent wind was from the south and south-west, atmosphere hot and oppressive in the afternoon, but often cool and pleasant in the morning, and generally dry.

During these warm days, from the 20th to the 27th, both serous diarrhœa and dysentery increased rapidly among young children, and to some extent among adults. The increase was so marked that nearly one-half of all the patients under my care, both in the office and out, were affected with one or the other of these diseases.

The 28th was cool and cloudy, with north-east wind. 29th and 30th, clear, cool, wind north and north-east.

From these detailed memoranda, as they were made from day to day, it appears that the first noted increase of bowel-affections for the present season was during the hot, damp, showery days of the 11th and 12th of June. It was during the night of the 11th, that I noticed the two first cases of severe vomiting, copious serous or rice water discharges, with muscular cramps, etc., in adults. One was a female, residing at 212 Kankakee Avenue, in the extreme southern part of the city, and the other was a man, on West Harrison Street, far towards the western border. From that date to the end of the month, ordinary attacks of diarrhœa and dysentery have continued to increase, especially among young children. But I have observed nothing unusual in their tendencies or results.

The only disease that has prevailed so extensively as to merit the title of epidemic, during the last three months, is measles. It first began to attract my attention on account of its unusual prevalence during the second week in June. It continued to prevail in all parts of the city through the remainder of the month. The cases were most numerous and severe between the 10th and 20th of June. Several adults suffered severe attacks, and in a few instances I found five and six cases in the same house, including the mother and all the children of the family. In a majority of the cases, the catarrhal symptoms were very severe; the eruption copious, and in some accompanied by sudaminæ. In a few of the cases of children under two years of age, lobular pneumonia supervened during the active stage. In two cases of nursing children, about the time for the decline of the eruption and fever, universal capillary congestion of the lungs supervened suddenly, giving to the lips, surface, etc., a dark purplish appearance, and causing speedily fatal results.

Coïncidently with the prevalence of measles, there occurred a considerable number of cases of scarlatina and whooping-cough. Between the 13th and 20th of June, several cases occurred, presenting the symptoms of a mild grade of cerebro-spinal meningitis, and in two the disease was more fully developed and proved rapidly fatal. One of these was a man who had recently arrived in the city from New Orleans, and was taken to the Mercy Hospital. The characteristic dark purple spots were thicker and more universally developed over the whole cutaneous surface than I had ever before seen. In addition to other remedies, the bromide of potassa was given to this patient liberally, but without any apparent effect. He died on the fourth day after admission to the hospital.

The influence of atmospheric conditions on the prevalence of certain diseases, is as clearly shown by comparing the mortality of the three past months with each other, as in the preceding details. Thus, in April, the gross mortality was 278, of which 13 only are attributed to diseases of the bowels; in May, the numbers are nearly the same; but in June, the gross mortality was increased to 319, of which 34 were attributed to cholera-morbus, diarrhœa, and dysentery. Should the special qualities of the atmosphere presented on the 11th and 12th, and from the 20th to the 28th of June, become the prevailing qualities during the month of July, the mortality from cholera, diarrhœa, and dysentery will be unusually great.

